# Oral Health Behavior Change in Mexican-American Caregivers: A Community-Based Intervention Study

**DOI:** 10.3390/ijerph16183409

**Published:** 2019-09-14

**Authors:** Nayanjot K Rai, Tamanna Tiwari

**Affiliations:** School of Dental Medicine, University of Colorado Anschutz Medical Campus, Aurora, CO 80045, USA; Tamanna.tiwari@cuanschutz.edu

**Keywords:** oral health knowledge, oral health behavior, self-efficacy, oral health prevention

## Abstract

An oral health prevention intervention was conducted with Mexican-American (MA) caregivers, focused on improving their oral health knowledge, behavior, and self-efficacy. Five in-person intervention sessions were conducted with caregivers, followed by a 15 min skill-building exercise. A goal-setting sheet was provided, and two goals were chosen for fulfilment during the three month intervention period. The data on parental oral health knowledge, behavior, and self-efficacy were collected pre- and post-intervention using a portion of Basic Factors Research Questionnaire (BRFQ). Paired t-tests were conducted to test significant differences in the means of pre- and post-intervention oral health behavior, knowledge, and self-efficacy scores, and pre- and post-intervention individual item scores. Forty six primary caregivers were enrolled. There were significant differences in the means of pre- and post-intervention oral health knowledge (*p* = 0.003), oral health behavior (*p* = 0.0005), and self-efficacy scores (*p* = 0.001). The individual item mean scores showed that there was a significant increase in the number of times caregivers checked for spots (*p* = 0.016) and a significant decrease in the consumption of sweet or sugary drinks (*p* = 0.032) post-intervention. Most of the caregivers believed that cavities were caused by germs in the mouth (*p* = 0.001), sharing utensils with children was bad for their teeth (*p* < 0.001), and fluoride toothpaste was best for a child’s teeth (*p* < 0.001). The intervention resulted in improved caregiver oral health knowledge, behavior, and self-efficacy.

## 1. Introduction

The role of parental behavior on oral hygiene and dietary control in reducing the prevalence and incidence of caries in young children is highly important [1]. Parents play a key role in setting a consistent oral hygiene routine for their children in the first years of life, which helps them to understand the importance of oral hygiene [2]. Parental attitudes and perceptions of the importance and value of oral health have been associated with the development of oral hygiene skills, including tooth brushing in children [3,4]. Parental perceptions of self-efficacy in certain behaviors, including brushing a child’s teeth, play an important role in good oral hygiene maintenance [5]. Parents with a stronger sense of efficacy set higher goals, persist longer at tasks, and perform with more effort, compared with parents with low self-efficacy [6]. Finlayson et al. (2005) reported that parents with higher self-efficacy, i.e., confidence in their ability to make sure that their child’s teeth were brushed at bedtime, were more likely to have their child brush at bedtime, compared with parents with lower self-efficacy [7].

For young children, positive parental behaviors and higher self-efficacy are essential for the maintenance of oral hygiene and reduced sugar consumption [1]. Douglass et al. (2001) found that children with parents supervising in tooth brushing had less dental caries prevalence as compared with children of non-supervising parents [8]. Within the Latino population, studies have found that parental knowledge, parental oral hygiene practices, and maternal untreated caries are some of the factors affecting Latino children under the age of 5 [8,9,10,11,12].

Children of Latino heritage experience significantly worse oral health outcomes in comparison with the general U.S. population, with the exception of American Indian children [13,14,15]. The Latino population constitutes one of the largest minority groups in the United States [16]. Latinos comprise a heterogeneous group that includes individuals from Mexico, Central and South America, and the Caribbean nations [17]. Although they have shared cultural values, individual and subgroup differences exist based on country of origin. However, most oral health promotion programs do not take into account these heterogeneities within Latino populations. Colorado has the seventh-largest Latino population in the United States. Latinos make up 21% of the state’s population, with 76% of Latinos of Mexican origin, who are highly concentrated in the Denver metro area [18]. Keeping the state’s demographics for the Latino population in mind, this oral health prevention intervention included only Mexican American (MA) caregivers. This study used a quasi-experimental design to test if an evidence-based oral health prevention intervention improved oral health knowledge, oral health behaviors, and self-efficacy in MA caregivers. In addition, the intervention focused on conducting skill-building for caregivers, to improve their confidence in maintaining their child’s oral hygiene.

Preceding this study, a needs assessment was conducted with MA caregivers in the Denver metro area, to develop a deeper understanding of the challenges faced by caregivers in oral health maintenance, and to understand the need for oral health prevention interventions in this community [19]. The needs assessment followed a Community Based Participatory Research (CBPR) methodology. The needs assessment highlighted that MA caregivers had some understanding of the etiology of dental caries, but prevention efforts were needed to reduce ECC in their children. They also reported a lack of confidence in performing oral hygiene for younger children, and quoted several barriers in seeking optimal care [19]. The present study was based on the results of the needs assessment and addressed the concerns of MA caregivers within the Latino community.

## 2. Materials and Methods

This study was approved by the Colorado Multiple Institutional Review Board (COMIRB) and conducted as an academic community partnership between the research team at the University of Colorado School of Dental Medicine and Servicios De La Raza, which is a Latino community serving organization located in the Denver metro area. The academic research depended on the community partner’s expertise in recruiting and connecting to primary caregivers (mothers, fathers, grandmothers) for participation in the intervention. The Servicios staff reached out to the community by contacting head starts and kindergartens in a bid to recruit participants for the study.

Forty-six primary caregivers, who had at least one child under the age of six, who self-identified as MA were recruited for the study. Information about the study was provided in both English and Spanish. A consent form that detailed the approach of the study was provided to the caregivers. A Spanish interpreter/translator was readily available to translate the consent form or general information.

### 2.1. Intervention Sessions

Five in-person intervention sessions were conducted by the Principal Investigator of the study with 7–10 caregivers present in each session. Each intervention session was of 50–60 min and utilized information on oral health using the cavity-free at three flipbook resource provided by the Colorado Department of Public Health and Environment (CDPHE) [20]. This flipbook is developed for the use of health care professionals and provides information on the importance of primary teeth, checking for white spots, the importance of seeing a dentist, not sharing utensils, the use of fluoride toothpaste, tap water, feeding practices, and preventive visits. Supportive guidance on goal setting was provided to the caregivers, for the purpose of behavior change, by the research coordinator/associate of the study. The goal-setting sheet [20] included nine goals, as follows: to provide regular dental care; to eat more fruit, vegetables, milk, and cheese; to brush with fluoride toothpaste; to drink tap water; to not share utensils with children; to not put baby to bed with a bottle; to wean baby off the bottle; to drink more water, and less juice and soda; and to only put water in a sippy cup. Out of these nine oral health behavior goals, the caregivers were asked to choose two that they wanted to accomplish during the 3-month period of the oral health prevention intervention. In addition to the goals, the caregivers were asked to define their confidence level in accomplishing the behavior change on a scale of 1–10, with 10 being the highest. The last 15 min of the session were dedicated to skill-building in the utilizing of the modified-bass technique for brushing and proper flossing techniques, to improve caregiver oral health self-efficacy. A 3rd-year dental student conducted this skill-building exercise.

### 2.2. Data Collection

The study used a quasi-experimental design, which included a non-randomized pre- and post-intervention evaluation. The pre- and post-intervention survey data were collected using a portion of the Basic Factors Research Questionnaire (BRFQ), which captures parental dental knowledge, attitudes, behaviors, and other psychosocial measures [21]. A paper-based survey in English or Spanish was given to the caregivers. The data were collected twice, once prior to the in-person session and once at the end of the study intervention period, i.e., after 3 months. The survey collected data on three measures: caregiver oral health knowledge, oral health behaviors, and self-efficacy.

Oral Health Behavior: Twelve items were used to obtain an overall behavior score representing the percentage of oral health behavior items answered with an “adherent” response. Adherent is the recommended oral health behavior, as defined by the study instrument.

Oral Health Knowledge: Fourteen items were used to obtain the overall knowledge score representing the total percentage of oral health knowledge items answered with an “adherent” response.

Self-Efficacy: Ten items were used to represent the overall self-efficacy scores. The self-efficacy measures maternal confidence in successfully engaging in recommended oral health behaviors for their child.

### 2.3. Phone Sessions

**Figure 1:**
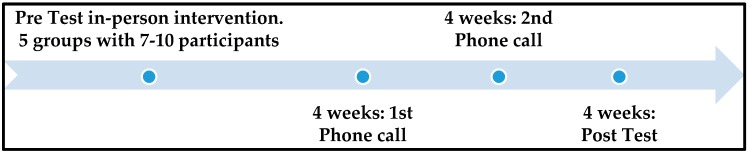
Study timeline over a 3-month oral health prevention intervention period.

Figure 1 explains the timeline of the study. All of the in-person intervention sessions were followed by two phone sessions. For each group, the first phone session was conducted 4 weeks after the in-person session, and the second was conducted 8 weeks after the in-person session. The phone sessions were scripted and included discussions on the goals chosen by the caregivers in the goal-setting sheets provided at the end of the in-person intervention sessions. The confidence level was also recorded during each phone session. All caregivers were telephoned according to their availability, provided to the study personnel responsible for recruitment. Each phone session lasted between 10 and 15 min. The phone sessions were conducted both in English and Spanish, based on the caregiver’s language of preference. The Spanish phone sessions were translated into English with the help of a Spanish interpreter.

### 2.4. Statistical Methods

Overall behavior and knowledge scores were calculated as a percentage of the individual items answered correctly (0–100). The self-efficacy scores were calculated as the overall score of the items answered correctly. Paired t-tests were conducted to see if there was any significant difference in the means of the pre- and post-intervention overall oral health knowledge, oral health behavior, and self-efficacy scores. Paired t-tests were also conducted to see if there was any significant difference in the means of pre- and post-intervention individual item scores. Another t-test was conducted to see if there was any significant difference in the confidence level means between day 1 and day 60 of the 90 day study period.

## 3. Results

A total of 46 primary MA caregivers were enrolled in the study. The response rate of the invited participants was 78 percent. Table 1 demonstrates the t-test results of differences in the pre- and post-intervention overall means for the three measures. There was a significant difference in the means of pre- and post-intervention oral health knowledge scores, indicating an increase in overall caregiver knowledge post-intervention (pre: 68.3, post: 78.2, *p*-value: 0.003). A significant difference in the means of pre- and post-intervention oral health behavior scores was also found, which indicated an overall improvement in the behavior of caregivers pertaining to their child’s oral health (pre: 48.2, post: 60.1, *p*-value: 0.0005). In the case of self-efficacy, the MA caregivers presented increased confidence in successfully engaging themselves in the recommended oral health behaviors for their child (pre: 8.2, post: 9, *p*-value: 0.001). Of the 46 participating MA caregivers, 36 responded to the confidence level question. They demonstrated a significantly higher confidence level at day 60 compared with day 1 (*p*-value: 0.002).

Table 2 demonstrates the significant paired t-test results for the individual items within the oral health behavior and oral health knowledge measures, pre- and post-intervention. In the case of oral health behavior questions, there was a significant increase in the number of times the caregivers checked for spots (pre: 31, post: 61, *p*: 0.016), and a significant decrease in the consumption of sweet or sugary drinks (pre: 83, post: 62, *p*: 0.032). A significantly higher number of children switched to drinking tap water post-intervention (pre: 44, post: 77, *p*: <0.001). In the case of the oral health knowledge questions, a significantly higher number of caregivers believed post-intervention that cavities were caused by germs in the mouth (pre: 55, post: 86, *p*: 0.001), sharing utensils with their child was bad for their child’s teeth (pre: 71, post: 94, *p* < 0.001), and fluoride toothpaste was best for their child’s teeth (pre: 68, post: 97, *p*: <0.001). There was also a significant increase in the knowledge of caregivers regarding the appropriate age for a child to brush his/her teeth by him/herself (pre: 24, post: 55, *p*: 0.026).

The focus of the phone sessions was to provide guidance and support to the MA caregivers, in the hope of achieving the desired behavior change and to overcome any possible challenges. The most common behavior change chosen by caregivers on the goal-setting sheet was “Eat more fruit, vegetables, milk and cheese” followed by “Keep germs to yourself”. Forty-seven percent of the caregivers chose to eat more fruits, vegetables, milk, and cheese, and 39 percent chose not to share their utensils with their children. The two behavior changes preferred the least were “Wean baby off bottle” and “Only water in the sippy cup”, which were chosen by only 11 percent of the caregivers. When examining the confidence levels, 53 percent of the caregivers showed an increase in their confidence level to accomplish behavior change.

The caregivers faced several challenges while achieving their goals, so several recommendations were provided, accordingly. The most common challenge was weaning baby off the bottle. Caregivers were advised to start diluting the milk with half water for the first few weeks, eventually eliminating the milk from the bottle. Some of the caregivers reported that they were successful in weaning their babies off the bottle and they were no longer drinking milk from the bottle. However, a few who had much younger children faced difficulties with the weaning process. Secondly, caregivers also faced difficulties in feeding vegetables to their children. In response, they were advised to puree the vegetables and incorporate them into meals, or to blend the vegetables into a smoothie. As a result, their children adapted well to these recommendations and started eating more fruit and vegetables. Thirdly, the caregivers reported that their children did not like the taste of tap water. They were advised to use filters on their taps, or to purchase bottled water containing fluoride. This facilitated exposure to fluoridated water, which eventually aids in preventing teeth from decay. The caregivers responded that the children rejected the filtered water, as they claimed the taste remained unaltered. Finally, it was challenging for the caregivers to avoid sharing utensils with their children. In response, they were encouraged not to use the same spoon as their children. They were advised to bring additional silverware to the family dining table. As a result, the caregivers reported that they were making a conscious effort to avoid sharing eating utensils with their children. However, they admitted that, on occasion, the eating utensils were shared amongst family members.

## 4. Discussion

The oral health prevention intervention resulted in an overall increase in MA caregiver oral health knowledge, oral health behavior, and self-efficacy. An increase in MA caregiver confidence levels in accomplishing their goals was seen post-intervention.

The oral health prevention intervention used cavity-free at three flipbooks by CDPHE, which served as a constructive means for increasing MA caregiver oral health knowledge, behavior, and self-efficacy. This flipbook has proven to be highly effective, and has served as a useful tool for providing oral health education and initiating positive behavior change in MA caregivers. It is an evidence-based educational resource, which has been used multiple times to educate Colorado-based populations. It is an excellent tool for teaching caregivers about the impact that nutrition, eating habits, fluoride toothpaste, and early dental visits can have on the oral health of their children.

The intervention session was conducted in a group format. Group learning fosters self-motivation via active learning and greater satisfaction in learning [22,23]. Group formats allow the participants to interact with each other, and to reflect on their own experiences as well as others in a way that engenders deep learning [24]. The group formats perform better in Latino communities, as previous studies have shown that Latino parents prefer interactive group sessions as they provide a supportive environment [25].

A goal-setting sheet was provided to the caregivers at the end of the in-person session. The caregivers were asked to choose two goals to accomplish during the 3 month intervention period. Goal-setting is one of the important elements in the cluster of behavior change innovations. Goal-setting increases the confidence of a person, directs his/her attention towards goal-relevant activities, and leads to skill-building on how to achieve goals; thereby, resulting in increased self-efficacy [26]. Goal-setting theory strengthens the objective that goals are an outcome to aim for and a reference standard for judging satisfaction [27]. Goal-setting exercises have helped parents of young children to bring about sustainable changes in oral health behaviors, which can contribute to reducing ECC in the long term [27,28]. All of the caregivers were telephoned 4 and 8 weeks after the in-person session, and were provided with feedback and encouragement on their progress. Patients/participants who set behavior change goals and receive follow-up through phone calls or emails perform better, as regular follow-up enhances sustained adoption of healthy behaviors [29].

The intervention session included a 10 min skill-building exercise on the modified-bass brushing technique and flossing, to improve parental self-efficacy. This may have promoted caregiver self-confidence in successfully engaging in recommended oral health behaviors, which increased post-intervention. Evidence-based motivational strategies around tooth brushing techniques are very minimal in literature. Research demonstrating the importance of maternal self-efficacy in maintaining a child’s oral hygiene supports the ideology of strategies that increase confidence in carrying out a child’s oral hygiene to be beneficial [30]. Studies have shown that teaching skill-building exercises to parents results in improvement in tooth brushing behaviors for their children, along with increased confidence ensuring brushing twice daily [3,25].

Oral health behaviors including the number of times the caregivers checked for spots, lowering the consumption of sweets and sugary drinks, and switching to tap water improved post-intervention. The more nuanced areas of knowledge including the use of fluoride toothpaste, the age at which children can brush their teeth alone, and the negative impact of sharing utensils with children also improved post-intervention. These results align with culturally tailored intervention studies that utilized similar oral health intervention programs conducted within Latino populations [25,27,31]. Such similarities in results suggest that Latino communities may not have detailed knowledge around the complex area of caries etiology [32]. However, the oral health prevention intervention in this study was able to enhance these complex areas of knowledge and behavior in the MA caregivers.

The limitations of this study include a relatively small and convenience sample size. This study also limits the generalizability of our results to Latino populations living at different geographic locations. Despite these limitations, this study makes a valuable contribution to the evaluation of oral health prevention intervention for MA caregiver oral health behavior change. Further research with a larger randomized sample, a control group, and a longer follow-up period is warranted.

## 5. Conclusions

This oral health prevention intervention program provided a framework for MA caregivers, in an effort to reduce caries incidence in children in the MA community where ECC is highly prevalent. The success of this intervention can be attributed to several components, including the use of a group format, an evidence-based intervention tool, time for feedback, and skill-building activities. We would be remiss not to admit that such interventions, although successful, are resource and labor-intensive.

## Figures and Tables

**Table 1:** Difference in the pre- and post-oral health prevention intervention overall means.

Variables (N = 46)	Pre-Score Means (SD)	Post-Score Means (SD)	Means (SD) *	*p*-Value
Oral health behavior ** (BH1–BH12)	48.2 (16.5)	60.1 (18.4)	11.86 (18.4)	<0.001
Oral health knowledge ** (KW1–KW14)	68.3 (15.5)	78.2 (15.2)	11.40 (21.8)	0.003
Self-efficacy *** (SE1–SE10)	3.4 (1.2)	4.2 (0.6)	0.77 (1.3)	0.001
Confidence level (n = 36)	8.2 (1.7)	9 (1.3)	0.79 (1.5)	0.002

* means and *p*-values obtained using paired t-tests. ** scores were calculated as the percentage of individual items answered correctly (0–100). *** overall score of the items answered correctly.

**Table 2:** Difference in the pre- and post-oral health prevention intervention variable means.

Variables (N = 46)	Pre-Score Means (SD)	Post-Score Means (SD)	Means (SD) *	*p*-Value *
Oral Health Behavior **				
BH1: Checked for spots	31 (0.46)	61 (0.48)	0.28 (0.71)	0.016
BH8: Consumption of sweet and sugary drinks	83 (0.37)	62 (0.48)	0.21 (0.57)	0.032
BH9: Drinking tap water	44 (0.49)	77 (0.42)	0.34 (0.69)	<0.001
Oral Health Knowledge **				
KW1: Cavities are caused by germs	55 (0.49)	86 (0.34)	0.32 (0.57)	0.001
KW4: Fluoride toothpaste is best for a child’s teeth	68 (0.46)	97 (0.16)	0.27 (0.45)	<0.001
KW13: Sharing utensils is bad	71 (0.48)	94 (0.23)	0.29 (0.46)	<0.001
KW18: Age for child brushing on its own	24 (0.42)	55 (0.49)	0.28 (0.75)	0.026

* means and *p*-values obtained using paired t-tests. ** scores for each variable were calculated as the percentage of correct answers marked by the participants.
